# Immune-related biomarkers in liquid biopsy for cancer: emerging tools for non-invasive precision oncology

**DOI:** 10.3389/fcell.2026.1878092

**Published:** 2026-09-14

**Authors:** Lai Wen, Xinhui Wang, Jinming Liu, Xinxin Pan, Yixin Zhang, Shuang Li, Mengying Tong, Yuankuan Jiang, Hewen Guan

**Affiliations:** 1 Laboratory of Integrative Medicine, First Affiliated Hospital of Dalian Medical University, Dalian, China; 2 Graduate School of Dalian Medical University, Dalian Medical University, Dalian, China; 3 Department of Dermatology, First Affiliated Hospital of Dalian Medical University, Dalian, China; 4 Department of Ultrasound, First Affiliated Hospital of Dalian Medical University, Dalian, China; 5 Dalian Dermatology Hospital, Dalian, China

**Keywords:** circulating tumor DNA, immune-related biomarkers, immunotherapy, liquid biopsy, therapeutic monitoring, tumor immune microenvironment

## Abstract

Liquid biopsy has emerged as a powerful non-invasive tool in precision oncology, providing real-time insights into tumor evolution, host immune responses, and dynamic changes in the tumor immune microenvironment. By enabling minimally invasive sampling, it can overcome several limitations of conventional tissue biopsy. This review summarizes the major biological sources and components of liquid biopsy, including circulating tumor DNA (ctDNA), circulating tumor cells (CTCs), exosomes, and circulating immune cells, and discusses their value as dynamic indicators of interactions during cancer immunotherapy. Particular attention is given to immune-related biomarkers associated with immune checkpoints, immunosuppressive mechanisms, and immune escape, including circulating immune cell populations, and inflammatory cytokine profiles. We further examine their potential applications in predicting treatment response, monitoring immune-related adverse events, assessing minimal residual disease, and detecting acquired resistance. In addition, recent technological advances that are accelerating the clinical translation of liquid biopsy are highlighted, including multi-omics integration, microfluidic platforms. These approaches have improved the sensitivity, accuracy, and multidimensional characterization of tumor- and immune-derived biomarkers. Nevertheless, biological heterogeneity, limited assay standardization, and the lack of large-scale prospective validation studies continue to restrict widespread clinical implementation. Overall, immune-related biomarkers detected through liquid biopsy offer considerable potential for the longitudinal monitoring of the tumor immune microenvironment and may improve non-invasive cancer diagnosis, therapeutic monitoring, and personalized immunotherapy in the era of precision oncology.

## 1 Introduction

Liquid biopsy, as an emerging non-invasive diagnostic technique, has shown significant potential in the field of precision oncology in recent years. Compared with traditional tissue biopsy, liquid biopsy enables real-time monitoring of tumor dynamics by analyzing components in blood and other body fluids, such as circulating tumor DNA (ctDNA), circulating tumor cells (CTCs), and extracellular vesicles (EVs) (Ma et al., 2024). With the widespread application of immunotherapy across multiple types of cancer, the development of reliable biomarkers for patient selection and treatment response prediction has become a critical issue that urgently needs to be addressed in clinical practice. These biomarkers can not only reflect the genomic characteristics of tumors, but also reveal dynamic changes in the tumor immune microenvironment.

At present, research on immune-related biomarkers in cancer liquid biopsy has progressively expanded beyond the detection of individual tumor biomarkers toward the comprehensive characterization of tumor–immune microenvironment interactions. Multiple liquid biopsy biomarkers in solid tumors can not only facilitate early diagnosis but also contribute to treatment response evaluation in patients with low levels of conventional tumor markers (Ma et al., 2024). Among these biomarkers, blood tumor mutational burden (bTMB) has emerged as an important indicator for predicting the potential benefit of immune checkpoint inhibitors (ICIs) (Barnett et al., 2024). A multidimensional liquid biopsy–based immune monitoring system can be established through the combined analysis of CTCs, ctDNA, exosomes, thereby improving the predictive accuracy of precision cancer immunotherapy (Wang et al., 2023). Currently, the development of liquid biopsy is shifting from single-biomarker detection toward an integrated analytical approach that combines ctDNA, exosomes, CTCs, immune cells, and multi-omics data, providing new opportunities for precision cancer immunotherapy and dynamic monitoring of treatment response.

Unlike existing reviews that primarily focus on categorizing individual liquid biopsy biomarkers, this review emphasizes the broader application of liquid biopsy in monitoring tumor–immune interactions and its expanding clinical utility in cancer management. Particular attention is given to immune-related biomarkers that can provide longitudinal information on both tumor progression and host immune responses. By integrating evidence from multiple liquid biopsy sources, including blood, cerebrospinal fluid, pleural effusion, ascites, urine, and stool, we provide a broader understanding of how different biospecimens capture tumor-associated and systemic immune information. In addition, we focus on the longitudinal application of immune-related biomarkers for patient stratification, response assessment, minimal residual disease surveillance, resistance detection, and immune-related toxicity monitoring. We further summarize emerging analytical technologies that facilitate sensitive and multidimensional biomarker detection, highlighting future directions for the clinical translation of liquid biopsy in precision immunotherapy.

## 2 Liquid biopsy specimens for detecting immune-related biomarkers

Liquid biopsy specimens originate from diverse biological compartments and provide complementary information regarding tumor characteristics and host immune status. Although blood remains the most commonly used source owing to its accessibility and ability to reflect systemic alterations, other body fluids, including cerebrospinal fluid, pleural effusion, ascites, urine, and stool, offer unique advantages in capturing local tumor microenvironments or organ-specific immune responses. Blood-based samples primarily reflect systemic tumor burden and immune activity, whereas anatomically specialized fluids may provide more direct insights into specific tumor niches. Collectively, these diverse liquid biopsy sources form a complementary framework for identifying immune-related biomarkers and understanding immune responses during cancer progression and treatment (Figure 1).

**FIGURE 1:**
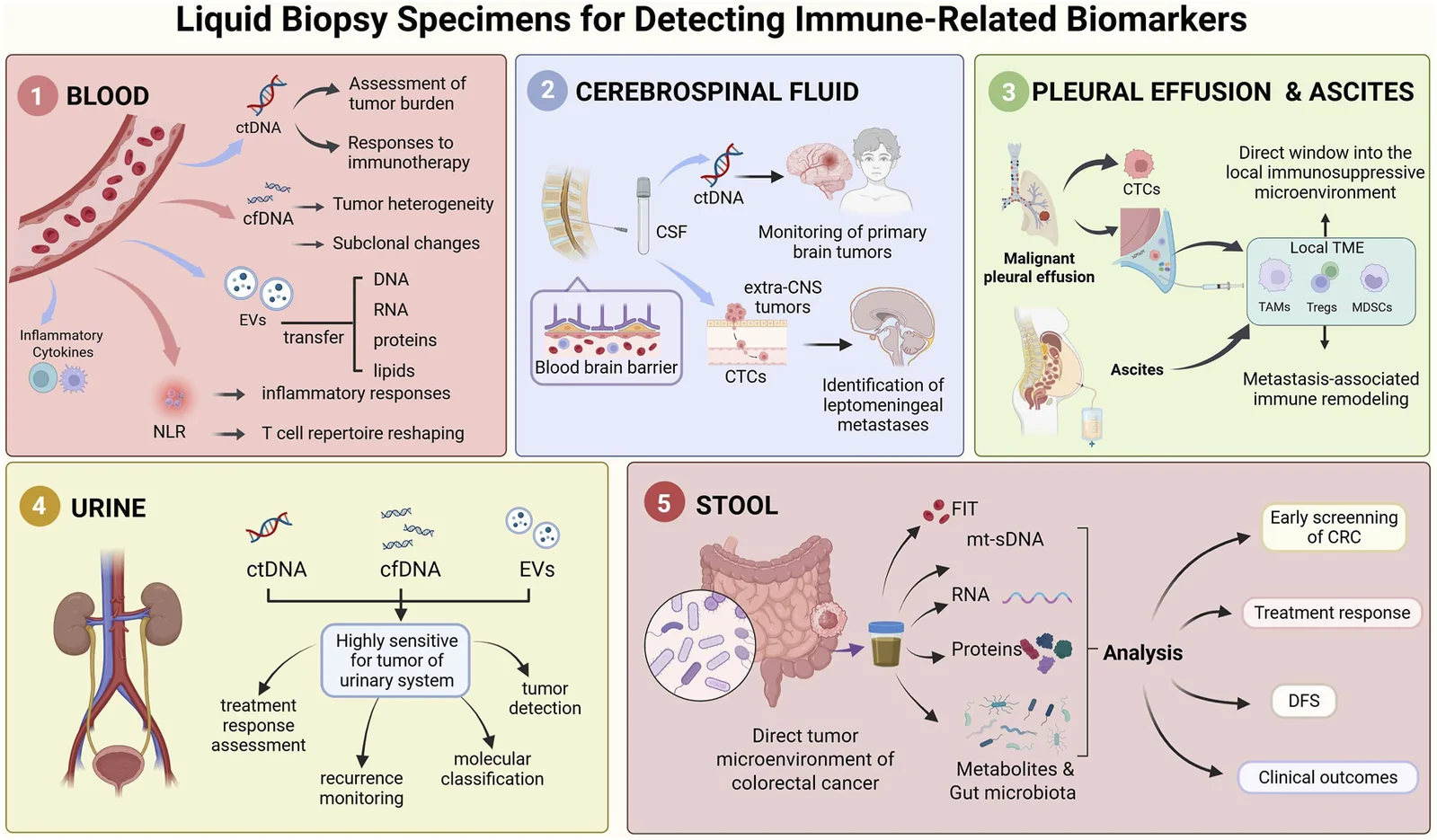
Liquid biopsy specimens for detecting immune-related biomarkers. (Created in BioRender. WEN, L. (2026) https://BioRender.com/7opae3d).

### 2.1 Blood

As the most widely used and clinically accessible source for liquid biopsy, blood provides a unique opportunity to simultaneously assess tumor-derived signals and host immune responses. Its major biological advantage lies in its ability to capture whole-body tumor burden, metastatic dissemination, and dynamic immune changes during treatment. Circulating DNA in blood consists of both ctDNA and cell-free DNA (cfDNA). ctDNA retains tumor-derived molecular signatures, enabling the evaluation of tumor burden, therapeutic response, minimal residual disease (MRD), and the development of acquired resistance. In comparison, cfDNA is mainly released during cellular apoptosis and necrosis and exhibits a relatively short half-life, making it suitable for dynamic monitoring of tumor heterogeneity and subclonal changes. In addition, cfDNA fragmentation patterns reflect tumor-associated epigenetic alterations, providing further insights into the molecular characteristics of tumors (Bartolomucci et al., 2025; Zhao et al., 2025; Gerratana et al., 2021). Blood contains abundant EVs released from tumor cells and other cell types. These vesicles carry transfer of DNA, RNA, proteins, and lipids, which reflect tumor status and intercellular communication (Liu et al., 2021; Liu and Wang, 2023). Longitudinal assessment of the neutrophil-lymphocyte ratio (NLR) provides insights into inflammation induction within the tumor microenvironment, dynamic tumor regression, and peripheral T cell repertoire remodeling associated with intratumoral immune responses (Hwang et al., 2022). The inflammatory tumor microenvironment is recognized as a critical determinant influencing cancer treatment outcomes. Circulating inflammatory cytokine levels not only serve as indicators of systemic inflammatory status but also represent effective immunomodulatory targets for cancer therapy. Several inflammatory cytokine-based gene therapies have already entered clinical trial stages (Lan et al., 2021). Together, these complementary biomarkers enable a multidimensional assessment of tumor–immune interactions and therapeutic responses during cancer progression and treatment.

### 2.2 Cerebrospinal fluid

Cerebrospinal fluid (CSF) is particularly valuable for liquid biopsy in central nervous system tumors, including gliomas and brain metastases. Because of the unique anatomical location of central nervous system (CNS) tumors, surgical biopsy is often associated with substantial risk. CSF-derived ctDNA liquid biopsy enables dynamic molecular monitoring and provides insights into the clonal evolutionary trajectories associated with the development of treatment resistance. Compared with peripheral blood, CSF is anatomically closer to intracranial lesions and is less affected by the blood–brain barrier. Therefore, CSF has a unique biological advantage in reflecting local neuroimmune status and dynamic changes within the intracranial tumor microenvironment (Berzero et al., 2023; Bettegowda et al., 2014). Especially in pediatric patients with primary brain tumors, CSF ctDNA detection has been associated with tumor burden, active disease, and leptomeningeal spread of disease (Miller and Karajannis, 2022; Seyhan, 2024; Miller et al., 2019; Miller et al., 2022). The clinical applications of CTCs in CSF have focused on their use as a diagnostic tool in patients with leptomeningeal metastases (LM) from solid, extra-CNS tumors (Diaz et al., 2024).

### 2.3 Pleural effusion and ascites

Pleural and peritoneal effusions, as common complications in patients with malignant tumors, are enriched with diverse molecular information derived from tumor cells and the tumor microenvironment, serving as valuable sources of tumor-associated biomarkers for liquid biopsy-based diagnosis, molecular profiling, and dynamic disease monitoring. Conventional diagnostic evaluation primarily relies on cytological examination of pleural or ascitic fluid to detect malignant cells. However, recent studies have revealed that pleural and ascites are enriched with CTCs and diverse tumor-derived molecular information, providing new avenues for precision cancer diagnosis, molecular characterization, and dynamic disease monitoring (Sorolla et al., 2021; Jung et al., 2016). Lung cancer-associated malignant pleural effusion-derived cfDNA displays distinct molecular characteristics compared with plasma-derived cfDNA, which may be primarily attributed to differences in genomic DNA fragmentation patterns arising from apoptotic and necrotic cell death pathways, together with the unique immune cell landscape shaped within the malignant pleural microenvironment. Moreover, single-cell analysis studies have demonstrated that malignant pleural effusions exhibit a distinct immune cell landscape with characteristic alterations (Huang et al., 2021). In ovarian cancer, IL-6, IL-8, IL-10, IL-1β, and IL-12 in ascitic fluid are considered key tumor-promoting mediators that directly or indirectly regulate immune cell populations, including tumor-associated macrophages (TAMs), regulatory T cells (Tregs), and myeloid-derived suppressor cells (MDSCs), thereby facilitating tumor progression and the establishment of an immunosuppressive microenvironment (Geng et al., 2023). Single-cell analyses have further revealed that ovarian cancer ascites exhibits a pronounced inflammatory microenvironment, with relatively high proportions of CD8^+^ T cells, macrophages, and dendritic cells (DCs), suggesting that ascites may serve as a valuable liquid biopsy specimen for characterizing the tumor immune microenvironment and its dynamic changes (Zheng et al., 2023). In gastric cancer-associated malignant ascites, T cells exhibit higher expression levels of immune checkpoint receptors, while CD8^+^ T cells display a terminally exhausted phenotype. Moreover, the levels of soluble immunosuppressive factors, including MMP-1, MMP-2, MMP-7, TGF-β1, HGF, E-cadherin, VEGF, and Ang-2, are elevated (Kim et al., 2025). Pleural effusion and ascites provide a more direct window into the local immunosuppressive microenvironment and metastasis-associated immune remodeling, as reflected by biomarkers such as CTCs, tumor-associated macrophages, T-cell subsets, and cytokine networks. Therefore, these body fluids have important value in investigating mechanisms of local tumor immune escape and treatment resistance.

### 2.4 Urine

Urine represents an important biospecimen for liquid biopsy, with considerable potential in early disease detection and longitudinal monitoring. Compared with conventional liquid biopsy samples such as blood, urine offers several distinct advantages, including non-invasive collection, convenient accessibility and repeatable sampling. Moreover, urine contains diverse disease-associated molecular components derived from the urinary tract and systemic circulation. Urinary cfDNA is generally more highly fragmented and, owing to the extremely short half-life of ucfDNA, is less stable than blood-derived cfDNA. Although urinary ctDNA is a promising biomarker, its limited stability and efficient isolation remain major challenges (Pisapia et al., 2025). In bladder cancer and urothelial carcinoma, urine-derived cfDNA, ctDNA and EVs have been investigated for tumor detection, molecular classification, recurrence monitoring, and treatment response assessment (Tse et al., 2021; Urabe et al., 2021; Tamura et al., 2024).

### 2.5 Stool

Stool samples are in direct contact with the colorectal mucosa and contain exfoliated tumor cells, microbial communities, local proteins, EVs, and metabolites, thereby providing a more direct and multidimensional representation of the colorectal tumor-associated intestinal environment and the early screening of colorectal cancer than blood. Currently, stool-based testing represents one of the most important sample sources for the early screening of colorectal cancer (CRC). The major stool-based biomarkers currently used include the fecal immunochemical test (FIT), RNA, proteins, metabolites, and the gut microbiota. Among these, the composition and characteristics of the gut microbiota have been shown to be closely associated with disease-free survival (DFS) in patients with colorectal cancer. Stool DNA testing can detect gene mutations and epigenetic alterations, including aberrant DNA methylation, thereby capturing tumor-specific molecular changes. Multitarget stool DNA (mt-sDNA) testing has also been used to screen individuals at high risk of colorectal cancer (Órdenes et al., 2023). Combining novel fecal biomarkers, such as fecal calprotectin (FC) and tumor M2 pyruvate kinase (TuM2-PK), with the FIT may further improve the sensitivity and specificity of colorectal cancer detection (Widlak et al., 2018; Ding et al., 2022). Emerging evidence suggests that stool-based gut microbiome profiles may help predict treatment response and clinical outcomes in colorectal cancer (Byrd et al., 2025; Huh et al., 2022).

## 3 Biological characteristics and dynamic alterations of immune-related biomarkers in liquid biopsy

Liquid biopsy enables the non-invasive evaluation of tumor progression and immune status through the analysis of diverse circulating components derived from both tumors and the host microenvironment in body fluids. As shown in Figure 2, the major components analyzed in liquid biopsy include ctDNA, CTCs, exosomal, and circulating immune cells, each of which plays a distinct role and has a different clinical application in tumor immune monitoring.

**FIGURE 2:**
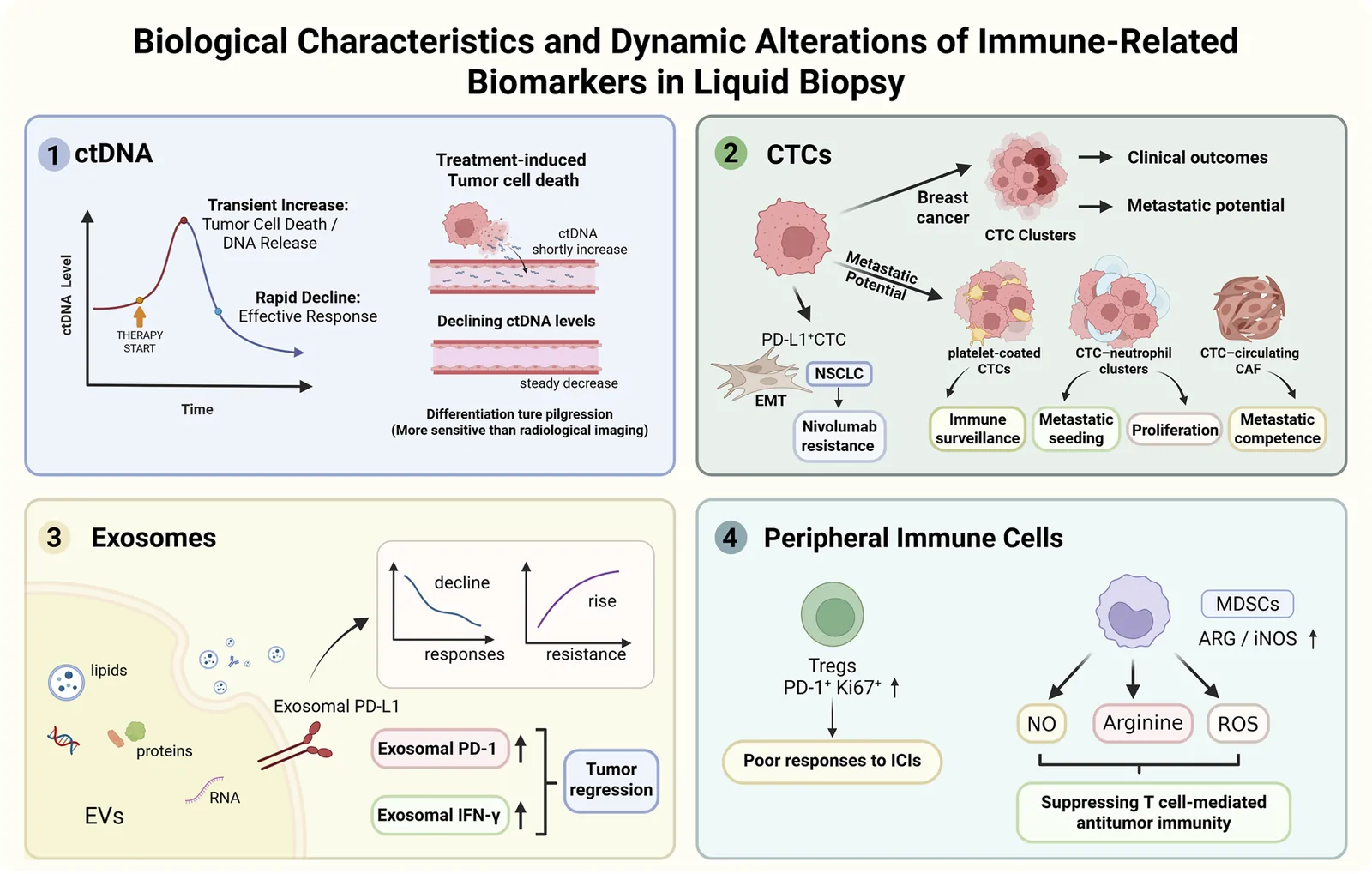
Biological characteristics and dynamic alterations of immune-related biomarkers in liquid biopsy. (Created in BioRender. WEN, L. (2026) https://BioRender.com/qa0y0cv).

### 3.1 ctDNA

Serial changes in ctDNA levels can be used to monitor minimal residual disease, treatment response, and disease progression. In several malignancies, including colon, breast, postoperative ctDNA clearance or persistently low levels are generally associated with a lower likelihood of residual disease and a reduced risk of recurrence (Bartolomucci et al., 2025; Shaw et al., 2024). Moreover, post-treatment changes in ctDNA are more sensitive indicators of therapeutic response than changes detected by radiographic imaging (Shaw et al., 2024; Goldberg et al., 2018; Tie et al., 2016). However, ctDNA should be interpreted in the context of treatment timing and modality. A transient increase in ctDNA may occur shortly after therapy because treatment-induced tumor cell death releases large amounts of tumor DNA into the circulation. In a clinical study of immunotherapy for non-small cell lung cancer (NSCLC), ctDNA response was defined as a reduction of more than 50% from baseline; changes in ctDNA occurred substantially earlier than radiographic responses and showed high concordance with imaging findings, while patients who achieved a ctDNA response had more favorable outcomes (Goldberg et al., 2018). In metastatic melanoma patients treated with anti–PD-1 antibodies, longitudinal ctDNA monitoring provides additional value in differentiating pseudoprogression from true disease progression, thereby addressing the limitations of conventional imaging-based evaluation (Lee et al., 2018).

### 3.2 CTCs

The dynamic changes in the number, phenotype, and molecular characteristics of CTCs can reflect tumor metastatic potential, immune escape status, treatment response. In metastatic breast cancer and prostate cancer, dynamic monitoring of CTCs enables the evaluation of treatment response, detection of disease recurrence, and prediction of patient prognosis (Hayes et al., 2006; Nakamura et al., 2024). In some patients, a renewed increase in CTC counts after an initial decline may indicate renewed tumor activity or emerging disease progression (Hayes et al., 2006). Notably, study has demonstrated that PD-L1-positive CTCs in NSCLC can exhibit epithelial–mesenchymal transition (EMT) phenotypes, which may be associated with potential resistance to the ICIs nivolumab (Raimondi et al., 2017). Through EMT, CTCs acquire enhanced metastatic and invasive capabilities, which represents one of the intrinsic mechanisms underlying immune evasion of CTCs in the peripheral bloodstream (Gu et al., 2024). CTC clustering is recognized as an adaptive mechanism across multiple cancer types. Compared with single CTC, CTC clusters exhibit enhanced metastatic potential and are associated with poorer clinical outcomes (Schuster et al., 2021). Heterotypic interactions between CTCs and non-malignant blood or stromal cells may further promote their survival and metastatic dissemination. For example, CTC–neutrophil clusters can enhance CTC proliferation and metastatic seeding, platelet-coated CTCs may protect tumor cells from immune surveillance, and CTC–circulating CAF clusters may increase metastatic competence (Schuster et al., 2021; Szczerba et al., 2019).

### 3.3 Exosomes

Exosomes are nanoscale membrane-bound EVs secreted by cancer cells. They contain a variety of biologically active cargoes, including proteins, DNA, RNA, and lipids, and play important roles in intercellular communication within the tumor microenvironment (Liu et al., 2021; Jin et al., 2022). Among exosome-associated immune biomarkers, exosomal PD-L1 has garnered substantial interest owing to its role in immune regulation and its potential value as a biomarker of response to immune checkpoint blockade. Longitudinal changes in exosomal PD-L1 may provide insights into tumor–immune interactions and adaptive immune-evasion processes during disease progression and treatment. In several clinical studies, decreases in exosomal PD-L1 during therapy, particularly at the time of on-treatment radiographic evaluation, have been associated with favorable responses, whereas persistent elevation or increases at later time points have been linked to non-response, disease progression, and poorer survival outcomes (Del et al., 2018; Akbar et al., 2022). In addition, exosomal immuno-oncological proteins and cytokines serve as important biomarkers for monitoring responses to ICIs, levated levels of exosomal PD-1 and IFN-γ indicate tumor regression (Chen et al., 2018).

### 3.4 Peripheral immune cells

Peripheral immune cells represent an important component of liquid biopsy and provide dynamic insights into systemic immune responses during cancer therapy. Persistent elevation of circulating Tregs, particularly activated or proliferating subsets such as PD-1^+^ and Ki67^+^ Tregs, may be associated with poor responses to ICIs and disease progression (Kang et al., 2022; Huang et al., 2024). However, the prognostic significance of Tregs may vary across biological compartments and tumor types; in malignant ascites from patients with ovarian cancer, poor prognosis has been associated with an increased ratio of CD8^+^ T cells to Tregs (Yoshida-Court et al., 2023). Myeloid-derived suppressor cells (MDSCs) are another major immunosuppressive population that can be longitudinally monitored during cancer therapy. Increased baseline levels of MDSCs, especially monocytic MDSCs, are associated with bad efficacy of immune checkpoint blockade therapy, whereas early declines may indicate favorable responses. Persistent or increasing MDSC levels may reflect sustained immunosuppression (Möller et al., 2024). The expansion of MDSCs is clinically correlated with the stage of solid tumors. Moreover, MDSCs exhibit markedly upregulated expression of Arg-1 and iNOS, which promotes the production of arginine, reactive oxygen species (ROS), and nitric oxide (NO), thereby suppressing T cell-mediated antitumor immunity (Möller et al., 2024; Zhang et al., 2015).

## 4 Clinical prospects of immune-related biomarkers in liquid biopsy

In the era of cancer immunotherapy, liquid biopsy–derived biomarkers have gained increasing clinical importance because they enable continuous monitoring of tumor evolution and dynamic changes in the immune microenvironment, unlike traditional tissue biopsy. Their central value lies in the ability to capture, in a dynamic, minimally invasive, and multidimensional manner, the real-time interactions between tumors and the host immune system, thereby providing critical insights for precision immunotherapy.

### 4.1 Pretreatment patient stratification and prediction of immunotherapy benefit

Certain liquid biopsy biomarkers have shown potential for identifying patients who are more likely to benefit from ICIs. Bratman et al. performed longitudinal monitoring of plasma samples from patients undergoing immune checkpoint blockade therapy and found that baseline ctDNA concentration was closely associated with progression-free survival, overall survival, and clinical response, suggesting that baseline ctDNA assessment may facilitate pretreatment patient stratification (Bratman et al., 2020). Peripheral blood-derived T-cell receptor (TCR) repertoires are critical tools for evaluating therapeutic responses in various solid tumors and are considered one of the key biomarkers for predicting responses to immune checkpoint inhibitor (ICI) therapy (Aran et al., 2022). Marked differences in fecal microbiome signatures have been observed between responders and non-responders to anti-PD-1 monoclonal antibody therapy. Moreover, in melanoma patients resistant to PD-1 blockade, fecal microbiota transplantation from responding donors drives the recipient microbiome toward a donor-like state, highlighting the gut microbiota as both a potential biomarker of immunotherapy response and a target for therapeutic intervention (Davar et al., 2021).

### 4.2 Longitudinal assessment of treatment response, tumor burden, and minimal residual disease

Serial analysis of liquid biopsy biomarkers provides a minimally invasive approach for tracking treatment response, changes in tumor burden, and molecular residual disease over time. Persistent or recurrent ctDNA detection during postoperative surveillance indicates post-treatment MRD or suggests an increased risk of clinical recurrence (Tie et al., 2016; Semenkovich et al., 2023). Similarly, studies have shown that CTC detection is an important approach for predicting tumor recurrence in patients with estrogen receptor-positive breast cancer (Sparano et al., 2018). Furthermore, emerging evidence suggests that tumor-derived exosomes may represent promising biomarkers for the longitudinal monitoring of MRD and therapeutic efficiency (Valcz et al., 2024).

### 4.3 Detection of immunotherapy resistance and guidance of treatment adaptation

Serial analysis of liquid biopsy components provides a minimally invasive approach for detecting primary or acquired resistance to ICIs and informing subsequent treatment adaptation. Failure to achieve ctDNA clearance during treatment was associated with radiographic response and survival outcomes in patients with advanced NSCLC, suggesting that early ctDNA assessment may help identify patients at increased risk of disease progression and potentially inform treatment intensification (Anagnostou et al., 2023). As a biomarker of molecular residual disease and recurrence, ctDNA can assist in evaluating the response to adjuvant immunotherapy in urothelial carcinoma and subsequently guide perioperative treatment strategies (Powles et al., 2021). Similarly, the presence of PD-L1-high-expressing CTCs is often associated with therapeutic resistance, and patients harboring PD-L1-positive CTCs demonstrate inferior responses to immunotherapy after 6 months of treatment in NSCLC (Tamminga et al., 2019).

### 4.4 Prediction and monitoring of immune-related adverse events

Liquid biopsy offers a minimally invasive strategy for predicting and monitoring immune-related adverse events (irAEs) during immune checkpoint blockade. Circulating cytokines, such as IL-17, as well as early changes in B-cell subsets, eosinophils have been associated with subsequent irAE development (Tarhini et al., 2015; Das et al., 2018; Zhou et al., 2023). Organ-specific biomarkers may further improve monitoring. For example, fecal microbiota profiles are linked to immunotherapy-mediated colitis, urinary CXCL-9 and TNF-αmay indicate ICIs-associated nephritis, and microRNAs from serum and EVs show potential for identifying ICIs-related pneumonitis (Chaput et al., 2017; Chavez et al., 2026; Farooqui et al., 2023; Deng et al., 2025). However, most biomarkers remain exploratory and require validation in larger prospective cohorts before routine clinical implementation.

## 5 Emerging technologies

As research in liquid biopsy and tumor immunology continues to advance, a variety of emerging technologies are rapidly being developed, opening new possibilities for the detection and characterization of immune-related biomarkers. Characterized by enhanced sensitivity, improved multidimensional integration, and superior dynamic monitoring capabilities, these technologies have the potential to overcome many of the limitations of conventional approaches in sensitivity, specificity, and informational complexity.

Ultra-sensitive sequencing increasingly combines deep coverage, error correction, and individualized variant selection to detect low-abundance ctDNA during immunotherapy. Schroeder et al. developed tumor-informed panels targeting up to 30 patient-specific mutations and applied UMI-based deduplication with approximately 6,000× sequencing depth, enabling sensitive multipoint tracking of ctDNA in melanoma (Schroeder et al., 2024). Microfluidic and biosensor-based platforms integrate target enrichment, molecular labeling, and signal readout within miniaturized devices, enabling rapid analysis of rare circulating particles and cells. Lu et al. developed a microfluidic system that enables on-chip integrated isolation of exosomes and PD-L1 analysis, thereby facilitating the assessment of exosomal PD-L1 protein biomarker abundance (Lu et al., 2022). Cai et al. developed a novel microfluidic-based platform for CTC isolation and enrichment, enabling direct analysis of CTC subtypes, surface-specific protein expression levels, and targeted mutations in specific genes (Cai et al., 2023). Together, these technologies enable integrated profiling of extracellular-vesicle- and CTC-associated immune markers, although broader prospective validation remains necessary.

## 6 Conclusion

In summary, liquid biopsy–based immune-related biomarkers have emerged as highly promising non-invasive tools in precision oncology, with broad applications in early cancer detection, prediction of immunotherapy response, dynamic disease monitoring, and prognostic assessment. By capturing not only tumor-derived molecular alterations but also dynamic changes in host immune status, liquid biopsy provides a more comprehensive view of the complex interactions between tumors and the immune system compared with conventional tissue biopsy. Despite these advances, several important challenges remain, including biological heterogeneity, temporal variability of biomarkers, limited detection sensitivity, lack of technical standardization, and insufficient large-scale clinical validation. These limitations underscore the importance of integrated multi-biomarker and multi-omics strategies. In particular, combined analysis of ctDNA, exosomes, cytokines, and circulating immune cells may improve predictive accuracy and strengthen clinical interpretability. With continued technological refinement, standardized workflow implementation, and large multicenter prospective validation studies, liquid biopsy–based immune biomarkers are anticipated to play an increasingly central role in dynamic precision immuno-oncology and personalized cancer treatment.
